# Increased acquired protease inhibitor drug resistance mutations in minor HIV-1 quasispecies from infected patients suspected of failing on national second-line therapy in South Africa

**DOI:** 10.1186/s12879-021-05905-2

**Published:** 2021-02-25

**Authors:** Adetayo Emmanuel Obasa, Anoop T. Ambikan, Soham Gupta, Ujjwal Neogi, Graeme Brendon Jacobs

**Affiliations:** 1 Department of Pathology, Division of Medical Virology, Faculty of Medicine and Health Sciences, Stellenbosch University, Tygerberg, Cape Town, 7505 South Africa; 2 Department of Laboratory Medicine, Division of Clinical Microbiology, Karolinska Institute, Stockholm, Sweden

**Keywords:** High-throughput sequencing (HTS), Boosted protease inhibitors (bPIs), Non-nucleoside reverse transcriptase inhibitors (NNRTIs), Nucleoside reverse transcriptase inhibitors (NRTIs)

## Abstract

**Background:**

HIV-1C has been shown to have a greater risk of virological failure and reduced susceptibility towards boosted protease inhibitors (bPIs), a component of second-line combination antiretroviral therapy (cART) in South Africa. This study entailed an evaluation of HIV-1 drug resistance-associated mutations (RAMs) among minor viral populations through high-throughput sequencing genotypic resistance testing (HTS-GRT) in patients on the South African national second-line cART regimen receiving bPIs.

**Methods:**

During 2017 and 2018, 67 patient samples were sequenced using high-throughput sequencing (HTS), of which 56 samples were included in the final analysis because the patient’s treatment regimen was available at the time of sampling. All patients were receiving bPIs as part of their cART. Viral RNA was extracted, and complete *pol* genes were amplified and sequenced using Illumina HiSeq2500, followed by bioinformatics analysis to quantify the RAMs according to the Stanford HIV Drug Resistance Database.

**Results:**

Statistically significantly higher PI RAMs were observed in minor viral quasispecies (25%; 14/56) compared to non-nucleoside reverse transcriptase inhibitors (9%; 5/56; *p* = 0.042) and integrase inhibitor RAM (4%; 2/56; *p* = 0.002). The majority of the drug resistance mutations in the minor viral quasispecies were observed in the V82A mutation (*n* = 13) in protease and K65R (*n* = 5), K103N (*n* = 7) and M184V (n = 5) in reverse transcriptase.

**Conclusions:**

HTS-GRT improved the identification of PI and reverse transcriptase inhibitor (RTI) RAMs in second-line cART patients from South Africa compared to the conventional GRT with ≥20% used in Sanger-based sequencing. Several RTI RAMs, such as K65R, M184V or K103N and PI RAM V82A, were identified in < 20% of the population. Deep sequencing could be of greater value in detecting acquired resistance mutations early.

**Supplementary Information:**

The online version contains supplementary material available at 10.1186/s12879-021-05905-2.

## Background

High-throughput sequencing (HTS) has unique advantages and significantly improves sensitivity in quantifying the minority HIV drug-resistant variants within HIV quasispecies [1]. Increased identification of pre-treatment minority drug resistance mutations (DRMs) compared to Sanger-based sequencing genotypic resistance testing (GRT) was reported from both resource-rich and resource-limited settings [2, 3]. The role of minority drug-resistant variants and their clinical consequences in the failure of combination antiretroviral therapy (cART) is debatable [4–18]. The presence of the minor variants remains unclear but clinical consequences cannot be ignored.

Studies have shown that even in adherent patients, those with pre-existing Y181C mutants have a triple higher risk of virological failure on an efavirenz-based cART regimen [19]. Several studies have shown that minority pre-treatment drug resistance was associated with reduced treatment efficacy for first-generation non-nucleoside reverse transcriptase inhibitors (NNRTIs), but not for rilpivirine and integrase inhibitors (INIs) [3, 17, 20]. In contrast, other studies have indicated that in a population with a relatively low prevalence of DRM, the use of deep sequencing to detect minority HIV-1 DRM has limited clinical benefit [21]. However, a study conducted by Inzaule et al., reported that incorporating the minor DRMs might improve the predictive value of GRT, but that very low thresholds of minority mutations can compromise the test specificity [22]. Data on acquired minority mutations on treatment-failure patients are limited.

In sub-Saharan Africa, South Africa has 23.6% pre-treatment drug resistance to efavirenz or NVP, followed by Namibia with 13.8%, while Zimbabwe has 10.9% resistance to NVP [8]. HIV-1 subtype C (HIV-1C) is the major HIV-1 subtype in South Africa, responsible for more than 90% of infections. The recommended second-line cART consists of the nucleoside reverse transcriptase inhibitors (NRTIs) zidovudine or tenofovir and lamivudine and a ritonavir-boosted (/r) protease inhibitor (PI), usually lopinavir (LPV/r) [23, 24]. Earlier studies from South Africa and Sweden reported that despite good adherence, there is an increased risk of virological failure in patients with HIV-1C on bPI-based regimens [20, 25]. Ex vivo and in vitro experiments also indicated large variations in susceptibility of HIV-1C viruses in the absence of PI resistance-associated mutations (RAMs) [26].

Studies have reported that the rates of virological failure on second-line cART are high in resource-limited settings, including South Africa, and are associated with the duration of exposure to previous drug regimens and poor adherence [27], mostly without any protease RAM [28]. In South Africa, with more than 4.5 million HIV-infected individuals accessing cART, approximately 145,000 (∼4%) are accessing second-line cART [29]. However, the drug resistance pattern in patients failing on bPIs is limited and often described by GRT through Sanger sequencing [30]. An earlier study with only seven patients indicated the presence of PI RAMs in bPI-failure patients, which was missed by bulk Sanger sequencing [31]. In clinical settings, Sanger bulk sequencing is the most common and widely used for HIV drug resistance testing. The limitation of the Sanger bulk sequencing method is that it can only detect variants with prevalence > 20% which is well known [32–34]. Studies have described the presence of minority HIV-1 drug resistance mutations in treatment-naïve patients which could potentially impact treatment outcome [4, 13, 16, 19, 35]. Next-Generation Sequencing (NGS) method have the unique advantage of detecting of minority variants with a threshold as low as 1%; although, this method can also generate errors, so when reporting low-frequency, caution should be exercised [36, 37]. Therefore, the primary aim of the present study was to determine the level and pattern of HIV-1 drug resistance in minor (< 20%) and major viral populations in patients receiving bPIs.

## Methods

### Ethics statement

The study was approved by the Faculty of Medicine and Health Sciences, Health Research Ethics Committee (HREC) Stellenbosch University, South Africa (N15/08/071). The investigations also complies with the South Africa National Health Act No 612003 and abides by the ethical norms and principles for research as established by the Declaration of Helsinki, the South African Medical Research Council Guidelines as well as the Department of Health Guidelines. A waiver of written informed consent was awarded to conduct sequence analyses on these samples by the Health Research Ethics Committee of Stellenbosch University, South Africa.

### Viral load

For HIV-1 viral load testing, we used the Abbott m2000sp and the Abbott m2000rt analyzers (Abbott Laboratories, Abbott Park, IL, USA). Viral RNA was isolated from patient samples according to the manufacturer’s instructions using the Abbott RealTime HIV-1 Amplification Reagent Kit.

### Study design

Convenient plasma samples were obtained from patients receiving bPIs as part of their treatment regimen (as referred by the clinician) with viral load > 900 copies/mL at the time of sampling from the diagnostic section at the Division of Medical Virology, Stellenbosch University, and the South African National Health Laboratory Services (NHLS), and were collected between March 2017 and February 2018 [20, 38]. We excluded patient samples with no previous cART regimen history and patients receiving first-line cART treatment regimens. Patients had their samples submitted for HIV-1 GRT to the NHLS. The NHLS provides routine genotypic antiretroviral drug resistance testing for clinics in the Western Cape, Gauteng and Eastern Cape provinces.

### PCR amplification and HTS

Reverse transcriptase PCR (RT-PCR), which consists of cDNA synthesis followed by first-round PCR, was performed using the SuperScript™ III One-Step RT-PCR System with Platinum™ Taq DNA Polymerase (Invitrogen/Life Technology, Cat. No. 12574026) using the primers 1810F (5′-GCTACACTAGAAGAAATGATGACAGCATG-3′) and 5220R (5′-CCCTAGTGGGATGTGTACTTCTGA-3′). The second-round nested PCR was performed with 2001F (5′-TGCAGGGCCCCTAGGAAAAAGGGCTGTT-3′) and 5087R (5′- ATCCTGTCTACYTGCCACACAAYC-3′) primers using the KAPA HiFi HotStart ReadyMix PCR kit. The amplified products were purified using the QIAamp gel extraction kit (Qiagen, Germany). For HTS, the purified amplicons were fragmented, and the library was prepared using NEBNext® Ultra™ DNA Library Prep Kit for Illumina® (New England Biolab, USA) with multiplexed NEB next adaptors. The samples were then pooled together with other unrelated non-viral indexed libraries. Paired end sequences of read length 250 bp were carried out on the Illumina HiSeq2500. The sequences are available in SRA (submission ID: SUB5871663).

### Bioinformatics analysis

The raw reads were adapter-trimmed using TrimGalore version 0.6.2, followed by the removal of the low-quality bases (Phred value score < Q30) by Sickle version 1.33. Duplicate reads were removed using FastUniq. The de novo assembly was performed using the Iterative Virus Assembler. The processed reads were aligned against individual *pol* gene sequences in very sensitive local mode using Bowtie2 in order to select reads originated from *pol* genes and create a consensus gene. The subtyping was performed using REGA version 3. The selected reads were then aligned against *pol* protein sequences using the BLASTX program from the BLAST package. The best BLASTX hit was chosen for each read for the amino acid counting, which was performed by in-house script. The resistance was interpreted as per the mutation lists provided in the Stanford HIVDB, accessed on 6 January 2019 [39]. The complete script is available in github: https://github.com/neogilab/MiDRMPol_SouthAfrica.

### Statistical analysis

Descriptive statistic like mean, standard division (for normally distributed data), median, interquartile range (IQR), frequency in percentage were performed in GraphPad Prism version 8 (GraphPad Software, CA, US). The association between categorical variables were performed using Fisher’s exact test. Comparison between two groups with continuous variable was performed with Students t-tests. The *p*-value less than 0.05 considered significant.

## Results

**Fig. 1:**
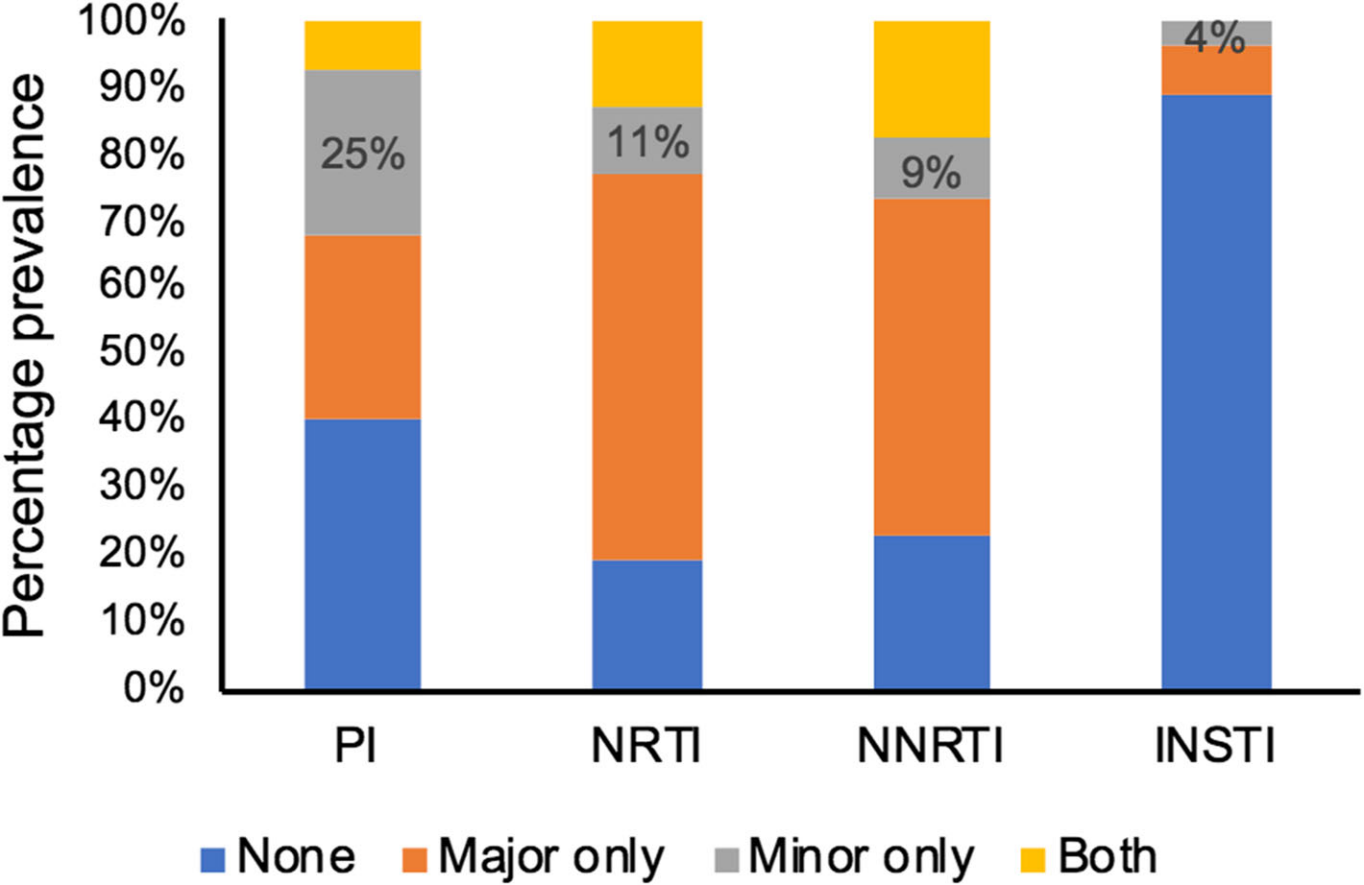
Percentage prevalence of PI, NRTI, NNRTI and INI RAMs in minor (< 20% of the population) and major (≥20% of the population) viral populations alone and together. The RAM in minor viral quasispecies are indicated

Among the 67 samples sequenced, current treatment regimen data were not available for 11 samples, and therefore they were excluded from further analyses. Among the 56 patients, 5.3% (*n* = 3) were on boosted ATV, while only one patient was on DRV/r and the rest (93%; *n* = 52) were receiving LPV/r. The median (range) viral load was 71,814 (937–5,500,000) copies/mL. HIV-1 subtyping identified 55 samples as HIV-1C and one as CRF02_AG. The NRTI, NNRTI and INI RAMs were observed among 25% (14/56), 57% (32/56), 50% (28/56) and 7% (4/56), respectively. Statistically significantly higher PI RAMs (25%; 14/56) were observed only in the minor viral quasispecies compared to NNRTI (9%; 5/56; *p* = 0.042) and INI RAM (4%; 2/56; *p* = 0.002). A total of 41% (23/56) did not have any PI RAMs (Fig. 1). The complete mutation profile is presented in the supplementary Table 1. There was no statistical difference in viral load (log_10_ copies/mL) in patients who only had PI DRM in minor population variants compared to patients who had only DRM in the major viral population [mean (SD): 4.92 (0.74) vs. 4.66 (0.88); *p* = 0.43].

**Fig. 2:**
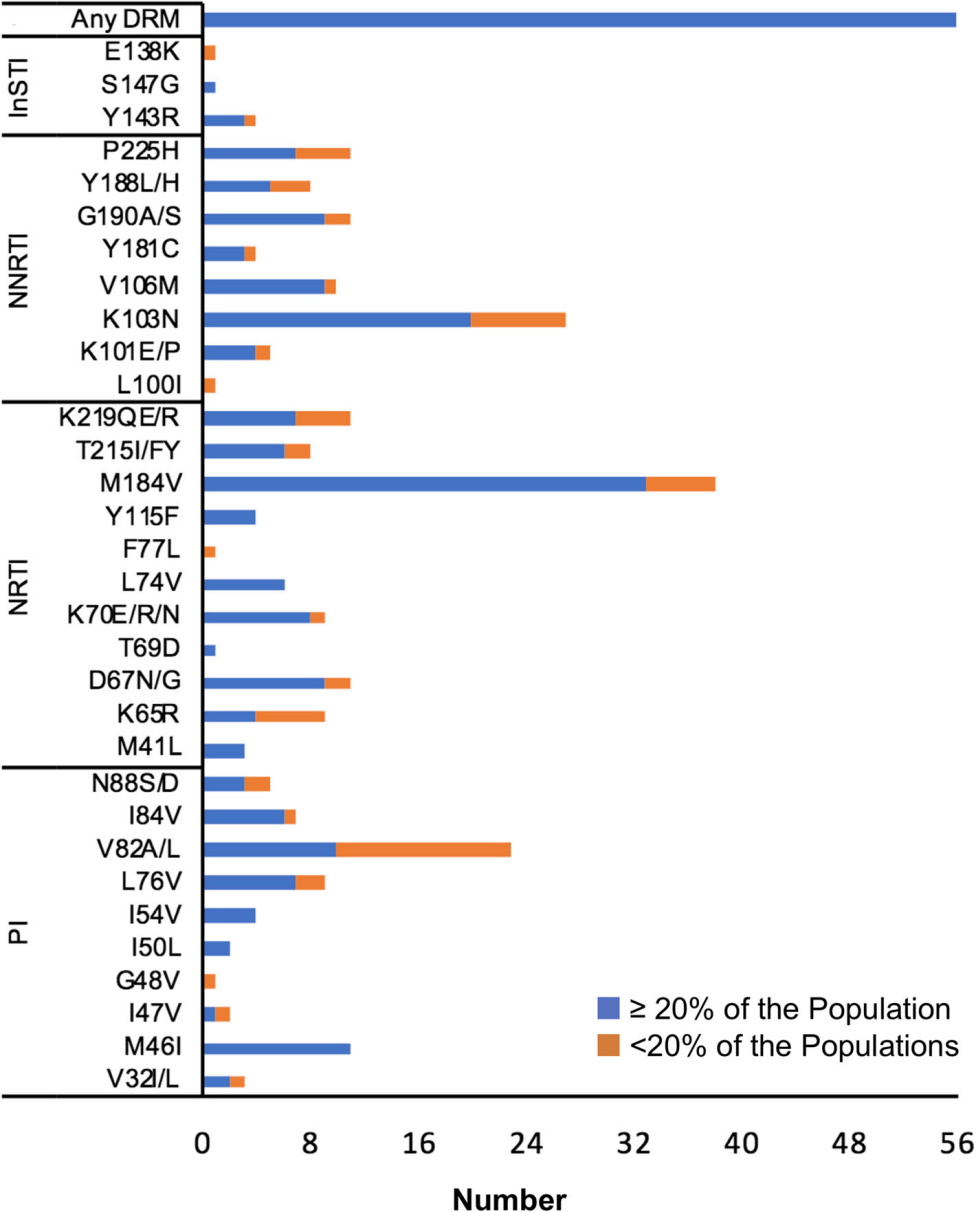
Number of different RAMs in minor (< 20% of the population) and major (≥20% of the population) viral populations

**Fig. 3:**
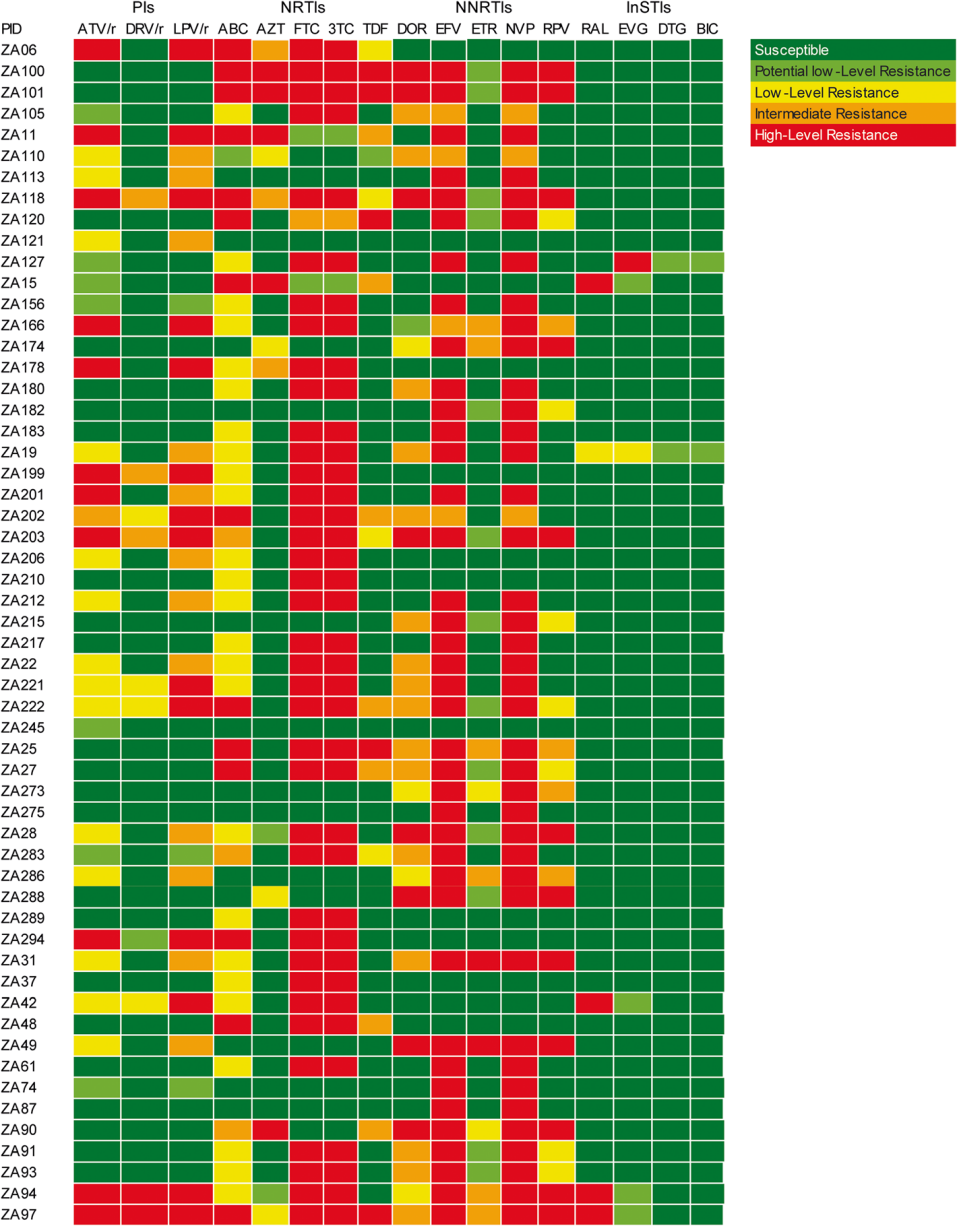
Predicted level of resistance to different antiretroviral drugs based on the Stanford HIVDB

All 56 patients harbored at least one DRM. Most of the DRMs in the minor viral quasispecies were observed in V82A mutation (*n* = 13) in protease and K65R (*n* = 5), K103N (*n* = 7) and M184V (n = 5) in reverse transcriptase (Fig. 2). Despite no mention of use of any INIs by the clinical reports, three patient sequences had the Y143R mutation in major viral quasispecies and one in minor viral quasispecies, which confers resistance to raltegravir (RAL). However, resistance to INI inhibitors was low in the settings. The predicted resistance pattern (as per the Stanford HIV Drug Resistance Database [HIVDB]) is given in Fig. 3. Half (28/56) of the patients had doravirine cross resistance. There were two patients (ZA94 and ZA97) who were resistant to all classes of drugs, indicating the presence of extremely drug-resistant HIV-1 strains in South Africa.

## Discussion

HTS assays have an intrinsic capability of detecting minority HIV-1 quasispecies mutation variants before they emerge as a majority variant under selection pressure, which might lead to virological failure. In this study we used HTS to type the DRM in both minor (< 20% of the population) and major (≥20% of the population) viral quasispecies and identified increased PI RAM in minor viral populations. Our study also indicated very low levels of transmitted INI RAMs in patients failing on a bPI-based regimen.

Earlier study have indicated that PI RAMs were uncommon in patients failing on second-line cART, with only 7% of patients on bPIs showing PI RAMs [40]. A study using Sanger sequencing indicated that 35% of the patients who had PI DRM failed on bPIs. A South African study conducted by Cohen et al. showed high-level resistance to LPV/r in 76/339 (22%), while 45/339 (13%) had high-level resistance to atazanavir and 2/339 (0.6%) had high-level resistance to darunavir [41]. Our study also showed that 34% of the patients had major PI RAMs in the major viral population, while 25% of the patients had PI DRM in the minor viral population. All the patients had at least one DRM. As most of the patients had high viral loads, there is a high chance of transmission of DRM if not treated timely. Furthermore, the PI RAMs in the minor viral population can evolve to become the majority under drug selection pressure.

Earlier studies indicated that HIV-1C has reduced susceptibility towards PIs without the emergence of major PI RAM [42] and that there is higher risk of virological failure [25]. Gag mutations can also confer reduced susceptibility towards PIs [43]. Furthermore, a study conducted in Nigeria reported PI RAMs in 62% of patients receiving PI-based second-line cART, with GRT being limited to patients with good adherence [44]. In East Africa, Inzaule et al. [45] found one or more major PI resistance mutations in 32% of unselected Kenyan patients with second-line ART failure and a median duration on PI-based ART of 3.1 years [45]. Few other observational studies in the African region have reported on ART exhaustion in 9 to 32% of patients failing second-line therapy [46, 47]. Clinical studies also reported that second-line failure was frequent in South African settings [30], which could further increase the chance of transmission of primary DRMs.

Even though there is no indication of the patients being administered RAL, three of the patients in our study harbored Y143R mutation in > 20% of the population. Previous studies on HIV-1C have shown major INI mutations at baseline in less than 5% of patients from Ethiopia (T66I, E138K, Q148R, and Q148H) and South Africa (Q148H, T66S, E92G, S147G, T66A, Y143YF and Y143H) [3, 20, 28]. However, the presence of INI RAMs in minor viral population is deemed not to have any clinical consequences [48].

## Conclusion

We show that the use of high-throughput resistance testing for GRT can greatly improve the identification of acquired PI RAMs in bPI-failing patients. Using HTS-GRT, PI RAMs (V82A) and RTI RAMs (K65R, M184V or K103N) were identified in < 20% of the population that Sanger-based sequencing failed to identify, strengthening their role in detecting the acquired mutations early. In resource-limited settings, the use of these high-throughput resistance-testing assays might help in the early detection of minor variants before evolving as a majority variant. Acquired drug resistance poses a significant threat to achieving the WHO/UNAIDS 90–90-90 targets for 2020. A recent WHO report also indicated an alarming surge in drug resistance across Africa [23]. Continuous surveillance, prevention, and monitoring at both minority variant and population-based level are critical to achieving the WHO/UNAIDS 90–90-90 target.

### Potential conflicts of interest

All authors declare no conflicts of interest.

## Supplementary Information

**Additional file 1 Table S1.** Individual patient wise mutation profiling.

## Data Availability

The raw FASTQ files dataset were deposited to NCBI-SRA. BioProject ID: PRJNA559799.

## Abbreviations

ART: Antiretroviral therapy
ATV: Atazanavir
BIC: Bictegravir
bPI: boosted protease inhibitors
BLAST: Basic Local Alignment Search Tool
cDNA: Complimentary Deoxyribonucleic acid
DNA: Deoxyribonucleic acid
DRM: Drug resistance mutations
DTG: Dolutegravir
EVG: Elvitegravir
GRT: Genotypic resistance testing
HIVDB: Human Immunodeficiency Virus Database
HTS: High-throughput sequencing
INI: Integrase inhibitors
INSTI: Integrase strand transfer inhibitor
NEB: New England Biolabs
NHLS: National Health Laboratory Services
NNRTI: Non-nucleoside reverse transcriptase inhibitors
NRTI: Nucleoside reverse transcriptase inhibitors
NVP: Nevirapine
PCR: Polymerase chain reaction
PI: Protease inhibitor
RAL: Raltegravir
RAM: Resistance-associated mutations
RNA: Ribonucleic acid
RT-PCR: Reverse Transcriptase Polymerase Chain Reaction
PCR: Polymerase Chain Reaction
WHO: World Health Organization
