# Supplementary material for: Increased acquired protease inhibitor drug resistance mutations in minor HIV-1 quasispecies from infected patients suspected of failing on national second-line therapy in South Africa
Source: BMC Infect Dis. 2021 Feb 25;21:214. doi: 10.1186/s12879-021-05905-2 (PMC7908688; doi:10.1186/s12879-021-05905-2)
Supplement: Supplementary file 1 — Additional file 1 Table S1. Individual patient wise mutation profiling. [file 12879_2021_5905_MOESM1_ESM.docx]

**Supplementary Table 1.** Individual patient wise mutation profiling

| **PID** | **PI_Major** | **NRTI** | **NNRTI** | **INI** |
| --- | --- | --- | --- | --- |
| ZA22 | V82A (1.13) | M184V (2.60) | K103N (1.63), P225H (1,66) |  |
| ZA174 |  | D67N (75.75), | L100I (1,025) |  |
| ZA212 | V82A (2.17) | M184V (99,88) | K103N (2.79), |  |
| ZA28 | V82A (1.15) | M184V (84.04), T215I (13.12) | K103N (2.53), K103S (1.27), V106M (1.08), Y188L (95.77), P225H (15.05) |  |
| ZA87 |  |  | K103N (1.31) |  |
| ZA 288 |  | D67N (8.98), | Y188L (99.75) |  |
| ZA 275 |  |  | K103N (99.38) |  |
| ZA 166 | M46I (93.84), V82A (99.18), I54V (99.6%) | L74 (99.28), Y115 (99.12), M184V (99.46) | Y181C (99.41) |  |
| ZA 180 |  | M184V (99.64), | K103N (99.52), P225H (99,51) |  |
| ZA 19 | V82A (17.68) | M184V (99.85), | K103N (98.09), P225 (99,34) | E138K (2,59) |
| ZA 201 | I50L (99.41), I54V (99.3) V82A (99.10) | M184V (99,67) | K103N (99,45) |  |
| ZA105 | N88D (1.38) | M184V (99.69) | P225 (99,07) |  |
| ZA11 | M46I (90.44), I54V (99.3), V82A (99.11) | M41L (81.34), D67G (81.14), K70R (81.87), L74I (81.40), T215 (99.51), K219E (99.59) | K103N (38.46)) |  |
| ZA118 | M46I (95.23), L76V (97.59), I84V (97.48) | D67N (99.60), K70R (99.41), M184V (99.77), K219Q (99.29) | Y188L (99.54) |  |
| ZA127 | N88D (2.77) | M184V (98.87) | K103N (98.58) | S147G (29.14) |
| ZA156 | V82L (96.78) | M184V (97,48) | K103N (97.31) |  |
| ZA202 | M46I (97.52), L76V (91.28), V82A (99.66) | K65R (99.51), M184V (99,89) | P225H (99.41), |  |
| ZA217 |  | M184V (99.77) | K103N (99.63) |  |
| ZA221 | L76V (34.33), V82A (12.27), N88D (33.25) | M184V (99.84) | K103N (99.54), P225H (99.63) |  |
| ZA61 |  | M184V (99.70) | K103N (99.48) |  |
| ZA91 |  | M184V (99.71) | G190A (99.25), V106M (41.11) |  |
| ZA93 |  | M184V (99.60) | V106M (41.11), G190A (99.57) |  |
| ZA94 | V32I (98.25), M46I (92.29), I47V (2.92), L76V (96.76), V82A (2.12), I84V (96.50) | M184V (99.65), T215F (99.10), | G190A (98.96), K101E (98.50) | Y143R (99.07) |
| ZA25 |  | M184V(99.63), K65R (98.78), Y115F (98.76), | K103N (99.47), V106M (98.99), Y181C (99.02) |  |
| ZA90 |  | M41L (98.60), T69D (98.39), T215Y (98.41), K219R (98.42), | G190A (99.25), V106M (99.36), Y188L (98.71) |  |
| ZA110 | V82A (10.34) | F77L (2.26), K219Q (37.05), | P225H (36.77) |  |
| ZA113 | V82A (1.82) |  | K103N (21.51) |  |
| ZA120 |  | K65R (9.26), L74V (61.11), Y115F (98.68) | K103N (98.58), G190A (99.42) |  |
| ZA182 |  |  | K103N (99.38), G190A (99,62) |  |
| ZA203 | M46I (98.86), I47V (93.55), L76V (97,69) I84V (97.20) | M184V (99.83), D67N (40.81), K70N (99.40), K219 (7.52) | Y188L (99.31), |  |
| ZA273 |  |  | K101E(99.73), K103N (99.69), |  |
| ZA49 | V82A(6.92) |  | K101E (76.26), K103N (22.46), Y181C (73.65), G190S (73.34), |  |
| ZA74 | M46I (88.2) |  | K103N (95.04) |  |
| ZA101 |  | K65R (3.81), D67N (8.27%), K70R (2.41%), M184V (5.69), T215I (97.68), K219E (2.63) | V106M (99.56), Y188L (1.61) |  |
| ZA222 | L76V (1.25), V82A (1.15) | M184V (1.12), K65R (1.26) | G190A (98.62), P225H (1,24) |  |
| ZA27 |  | M184V (2.11), K65R (1.09) | K103N (1.87), K103S (95.78), V106M (1,24), G190A (97.46) |  |
| ZA37 |  | M184V (1.10) |  |  |
| ZA 199 | M46I (96.51), M46L (1.16), L76V (58.73), I84V (62,88) | M184V (99.96), L74V (99.83), Y115F (99.79), |  |  |
| ZA06 | M46I (95.36), I54V(99.4), V82A (99.48) | D67N (99.38), K70R (99.51), M184V (98.60), K219Q (99.34) |  |  |
| ZA15 | I50L (99.82) | M41L (81.34), D67G (81.14), K70R (81.87), L74I (81.40), T215Y (99.51), K219E (99.59) |  | Y143R (99.47) |
| ZA178 | M46I (92.37), G48V (1,19), V82A (99.12) | D67N (99.45), K70R (99,36), M184V (99,06), |  |  |
| ZA206 | V82A (99.01) | M184V (99.43) |  |  |
| ZA210 |  | M184V (76.70) |  |  |
| ZA283 | V82L (2.16) | D67N (99.05), K70E (99.38), M184V (99.70) | Y188H (4.95), V106M (99.28) |  |
| ZA289 |  | M184V (99.70) |  |  |
| ZA294 | M46I (90.51), V82A (99.20), I84V (61.54), N88S (37.85) | M184V (99.80), L74V (99.64) |  |  |
| ZA31 | V82A (1.10) | M184V (99.61) | K101P (97.05), K103N (1.70), K103S (97.85), P225H (1.49) |  |
| ZA42 | V32L (42.74), L76V (93.86), V82A (99.52), I884V (99.52) | M184V (99.78) |  | Y143R (99.07) |
| ZA48 |  | K65R (99.14), M184V (99.70) |  |  |
| ZA 286 | V82A (31.26) |  | K103N (29.17), Y181C (10.43) |  |
| ZA100 |  | K65R (1.85), D67N (97.8%), K70R (97.92%), M184V (99.73), T215I (97.68), K219E (97.81) | V106M (99.56), Y188L (1.61), |  |
| ZA121 | V82A (5.32) |  |  |  |
| ZA183 |  | M184V (99.52), K219Q (2,04) | K103N (15,39) |  |
| ZA215 |  |  | K103N (30.88), V106M (8.39), G190A (11,69) |  |
| ZA245 | N88D (39.19) |  |  |  |
| ZA97 | V32I (1.57), L76V (1.39), I84V (1.47) | K65R (98.29), M184V (99.84), T215F (1.33), | K101E(1.12), K103N (98.19), G190A (1.34), P225H (97.88), | Y143R (1.23) |
